# Oral anticoagulation with vitamin K inhibitors and determinants of successful self-management in primary care

**DOI:** 10.1186/s12872-016-0326-z

**Published:** 2016-09-13

**Authors:** E. Tamayo Aguirre, A. Galo-Anza, O. Dorronsoro-Barandiaran, E. Uranga-Saez del Burgo, A. Ostiza Irigoyen, A. Garcia-Carro, I. Lopez-Fernandez, N. Colera, P. Saez-Garbayo, I. Tamayo-Uria

**Affiliations:** 1 Gros Health Centre, Donostia, Gipuzkoa, Spain; 2 Primary Care Research Unit-Gipuzkoa, Osakidetza, Spain; 3 Red de investigación en servicios de salud en enfermedades crónicas (REDISSEC), Centro de investigación en cronicidad Kronikgune, Instituto Investigación Sanitario Biodonostia, Osakidetza, Spain; 4 Pasai Donibane Health Centre, Gipuzkoa, Spain; 5 Usurbil Health Centre, Gipuzkoa, Spain; 6 Egia Health Centre, Donostia, Spain; 7 Lasarte Health Centre, Gipuzkoa, Spain; 8 Lezo Health Centre, Gipuzkoa, Spain; 9 Amara Health Centre, Gipuzkoa, Spain; 10 ISGlobal Barcelona Institute for Global Health, Barcelona, Spain; 11 Universitat Pompeu Fabra (UPF), Barcelona, Spain

**Keywords:** OAT, Self-management, Access, Abilities, Time in therapeutic range, Quality of life

## Abstract

**Background:**

Self-management may be an option to monitor oral anticoagulant therapy in health systems, but before recommending it, we need to assess patients’ ability to take on this task. The purpose of the study was to describe patients’ ability to self-manage and associated factors.

**Methods:**

This was a 3-year prospective quasi-experimental study with a control group. Overall, 333 patients on anticoagulant therapy from seven primary care health centres of the Basque Health Service were included in the intervention group and followed up for 6 months after the intervention, assessing their ability to self-test and self-manage. The intervention consisted of a patient training programme, providing detailed information on their condition and its treatment, and practical training in how to use a portable blood coagulation monitor and adjust their anticoagulant dose. Comparisons were made with a control group (333 patients receiving OAT under usual care from the same seven health centres).

Outcome variables were ability to self-manage, quality of the outcome (in terms of time in therapeutic range), and quality of life in the intervention group, and general patient characteristics (age and sex), clinical variables (reason for OAT, INR range), and quality of the outcome (in terms of percentage of INR measurements in range and complications) in both groups.

**Results:**

Overall, 26.13 % of patients invited to participate in the intervention agreed. Of these, 99 % successfully learned to self-manage their OAT. Just 4.2 % did not complete the follow-up, in all cases for reasons unrelated to self-management, and 4.5 % required additional learning support. Outcomes were better than under usual care in terms of percentage of INR measurements in range (12 %), rate of complications (4 %) and quality of life (9.2 %).

**Limitations:**

Patients were only followed-up period for 6 months and the study was conducted in a single health organization. Though patients eligible to participate were selected randomly, they were not randomly allocated to the groups. This is a potential source of selection bias. Data needed to calculate in-range time were not collected from controls; rather the results for the self-management group were compared with external data from other studies.

**Conclusions:**

Almost all participants achieved competency in self-management, with no differences by age, sex, concurrent illnesses, polypharmacy or educational level. The greatest barrier to self-management was the attitude of patients themselves and those around them. Self-management in primary care is a good alternative to usual care, patients having longer times in therapeutic range and fewer complications, and improving their quality of life. Remote management is a good support tool.

**Trial registration:**

ClinicalTrials.gov Identifier: NCT01878539.

## Background

Oral anticoagulant therapy (OAT) with vitamin K inhibitors (acenocoumarol, warfarin) and rivaroxaban, apixaban and dabigatran (new anticoagulant drugs) is a preventative therapeutic measure for the treatment of cardiovascular diseases, such as atrial fibrillation (the most common use), valvular diseases and venous thromboembolic disorders. Its use has progressively increased in recent years [1, 2]. In our study, we only considered the first type, namely, vitamin K inhibitors.

In the aforementioned cardiovascular diseases, there is a high risk of thromboembolism and OAT has shown to be effective in the prevention of associated complications, although it also increases the risk of bleeding [3, 4]. Approximately 1.7–2 % of the Spanish population are on this type of treatment, the figure reaching up to 9–10 % in those over 65 years old, and increasing by 10 % per year [5]. The mean age of the population continues to rise and, with it, the prevalence of chronic illness; as a consequence, haematology services are becoming overloaded and primary care providers have had to become involved in the monitoring and follow-up of patients requiring OAT. The efficacy of OAT is related to maintaining the patient within a certain INR range (commonly between 2 and 3) [6]. Often, however, the INR strays outside this range (35–40 % of cases) [1–3, 5], and this is associated with new cardiovascular events, such as thrombosis due to low INR or bleeding due to high INR [3, 7, 8]. All the publications identified in the literature report high percentages of INR measurements being outside the target range, and hence, regular monitoring is necessary. This can be considered one of the weaknesses of OAT [3, 6], and is associated with new cardiovascular events, such as thrombosis due to low INR or bleeding due to high INR [3, 7, 8].

In the optimisation of INR control, patients play an essential role. For this reason, as in other chronic conditions, studies have been conducted exploring INR control with patient self-management [9, 10]. Self-testing is defined as patients using the coagulometer and calculating the INR themselves; they then submit the results to health professionals, who decide on the appropriate regimen and the date for the next check-up. Self-management is patients adjusting the treatment themselves as a function of their INR self-test results.

The efficacy of self-management of OAT has been assessed in multiple clinical trials, and their results have been synthesized in a systematic review [11]. This review indicates that self-management patients obtain similar or better results (data varying between authors) than patients under usual care, those under self-management keeping their INR in the therapeutic range for longer, and having significantly lower rates of thromboembolic and haemorrhagic complications [12–14]. The associated increase in the number of tests may also be a factor leading to better outcomes (more frequent tests making it easier to identify INR deviations early and promptly adjust medications, increasing the time in-range) [15–17].

The studies included in the review excluded a variable proportion of patients, due to clinical condition, age, level of education, ability to learn and/or the opinion of clinicians, with specific profiles emerging as a function of age, functional and cognitive status and underlying condition [11, 18]. These studies identify multiple advantages of self-management including reductions in the time dedicated to monitoring, both by clinicians and patients, in costs, and in the inconvenience associated with regular testing; as well as improvements in quality of life and satisfaction of patients [19].

When assessing the feasibility of introducing self-management in routine practice in primary care, it is first necessary to identify which patients are candidates for this type of care and associated factors [20]. According to Jefferson Medical College (2005), potential candidates are patients requiring long-term anticoagulation therapy, who are highly motivated, and have sufficient manual dexterity and vision [17]. Involvement and education of patients in OAT, in terms of knowledge of their condition and its treatment and management, have been recognised as factors contributing to achieving good outcomes using this treatment model [3]. Further, the use of new information technologies (Internet, e-mail, SMS) may favour patient self-management [21], and this should be taken into account. Self-management is not currently part of routine practice with isolated exceptions.

Taking into account the current situation, the objective of this study was to explore factors related to self-testing and self-management abilities of patients on OAT in primary care, as well as assess the quality of the outcome achieved under this type of treatment [21, 22] and the clinical consequences, comparing with usual care through health centres and haematology services.

## Methods

### Study design

This was a multicentre prospective controlled quasi-experimental study conducted between March 2012 and November 2014 to identify factors associated with the ability to self-manage and the clinical consequences of self-management in OAT patients. The study protocol was published by Tamayo-Aguirre et al. [22].

We included patients over 16 years of age on OAT (for any reason) from seven different primary care health centres in rural or urban settings of an integrated healthcare organisation (Donostialdea). The overall organisation had a catchment population of approximately 324,000, and 7,213 patients were on OAT (slightly over 2 % of the total population). We excluded patients with serious diseases (hospitalised, or with a terminal diagnosis) and physically or mentally disabled individuals who lacked adequate caregiving, as well as those who had been on OAT for less than 1 year.

To estimate the appropriate sample size, we considered that for studies of predictive models, it has been established at least 10 events of the dependent variable of interest are needed for each independent variable included in the multivariate logistic regression model [23, 24]. We estimated that we needed at least 100 dependent variable events. Previous studies [7, 12] indicate that approximately 50 % is a “suitable” percentage, and hence we would need 100 suitable and 100 non-suitable candidates, yielding a total of 200 patients. In the study published by Fitzmaurice et al. [10], 24 % of patients declined to participate, 7 % did not attend study meetings, 6 % did not sign the informed consent form, and 3 % were excluded by researchers, amounting to a 40 % loss to follow-up. Assuming then that around 60 % of patients agree to participate in this type of intervention, if we wanted 200 participants in the intervention group, we estimated we would need to recruit at least 333 patients for the initial sample, and for comparison, the same number of controls.

### Patient selection and recruitment

We spread awareness of the project among participating organisations and members of staff potentially affected were informed, with meetings in the form of clinical sessions in health centres. Based on anonymised lists of patients under active anticoagulant therapy at participating centres, the coordinating research unit randomly selected a sample of patients, who were then contacted by their assigned primary care clinicians and invited to participate in the study. Those who agreed to join the intervention group were given a first appointment with a member of the research team who provided them with information regarding the study, requested their written informed consent to participation in the programme and allocated them to a training group. Those who declined to participate in the intervention were invited to participate as controls. They were provided with information about the study, and asked for oral consent, which was considered sufficient as we only needed to access data from their records (no intervention); further, we obtained approval from Osakidetza to use data from the electronic health records.

### Description of the intervention

We designed a bilingual (Spanish and Basque) training programme focused on understanding and application of self-management techniques. In this programme, participating individuals received training on OAT, self-testing and self-management (Table 1). The training was based on a programme developed in the regions of Aragón and Catalonia, with written support materials (on a course of oral anticoagulation) provided by Roche, and was adapted to the needs of our study. It started with a workshop in which specialised nurses provided theoretical and practical information on OAT, and subsequently, four workshops were held with each patient individually, in which he/she learned to use the CoaguChek XS coagulometer, interpret INR results, adjust doses and enter related data in the online monitoring application (for more details, see Appendices I and II).

**Table 1:** Timetable and content of the training

Workshop	Duration	Type	Number of patients	Content
1	1 h	group/individual	2	OAT, starting self-management
2	1 h	individual	1	Coagulometer, tables, Internet
3	30 min	individual	1	Checking, clarification, survey
4	30 min	individual	1	Data entry, starting point
5	1 h	Individual	1	End of study, survey

### Study variables

The primary outcome variable was the capacity of patients in the intervention group to self-manage. This was assessed at the time of each INR measurement, considering whether they had taken decisions following the recommendations given (depending on the INR value obtained, the recommendation was to repeat the measurement, increase, maintain or decrease the dose, or contact the doctor) and reported the test result (Appendix II). This was based on the daily telemonitoring by the clinician of the actions of the patient. Patients were considered able to self-manage (positive outcome) if 95 % of their actions were correct (according to the recommendations given) and they committed no serious errors (greater than 20 % deviation from the recommendations as specified in Appendix II).

Patients were checked on every day through the web service (97 %) or over the telephone (an option used by 3 % of patients), and at the end of the study period (6 months), data collected in the coagulometer were retrieved and compared with those supplied directly by the patients.

For all participants, we collected data on personal and clinical characteristics, risk factors, all current medications including anticoagulants, time on OAT, and recommended INR range, as well as any vascular complications that developed and INR control in terms of percentage of INR measurements in range over the study period. In intervention group patients, we also assessed time in therapeutic range and quality of life (see below), comparing baseline (when they had been under usual care) and the period under self-management. These data were collected either from the patient him/herself or from the electronic health records of the Basque Health Service. In addition, we collected basic information on individuals who declined to participate (age, sex, current medications, vascular complications and INR control).

### Follow-up

Health records of control group patients were reviewed at 6 months after their inclusion in the study. In the case of intervention group patients, once we had checked the ability of a patient to self-manage, he/she started to do so and was then followed up for 6 months (i.e., from the end of the training until the end of the study).

During the follow-up period (6 months), no meetings were scheduled with these participants. To minimise risks to patients and reassure researchers, data provided by patients remotely (over the web or telephone) were reviewed, and there were no cases in which their level of control was sufficiently poor to indicate a need to intervene. Given the possible need for support, two channels of communication were provided:

1. Telephone: access to a 24/7 helpline to address both technical and clinical queries
2. Internet: entering of self-management data (namely, treatment variables, dose changes decided, any clinical incidents occurred or other comments, and the date of the next check-up) into a form on a website (https://autocontrol.taonet.es/tao). The data on this website was reviewed daily by a doctor, with the single goal of assessing whether patients were at risk.

To assess the impact of the intervention in terms of quality of life and patient satisfaction, we carried out a survey based on the questionnaire of Sawicki [13] designed to assess quality of life in OAT patients in Germany, previously adapted to the Spanish setting by Sánchez González [23] and used by Dávila Blazquez [25]. The questionnaire consists of 32 questions grouped to assess five parameters. Intervention group patients were asked to complete questionnaires at baseline and again at 6 months after the intervention.

### Statistical analysis

On the one hand, we created a basic profile of patients who declined to participate in the study with sex and age data. On the other, for the intervention and control groups, we considered a wider range of information: reason for OAT, INR range, length of treatment, risks factors, complications before and during the study, polypharmacy, and whether they were independent for activities of daily living or had a caregiver. Using this, profiles of intervention and control patients were created and compared, to determine whether they were significantly different, using the Student’s t and the chi-square tests for numerical and categorical variables, respectively.

We then wanted to assess the results of self-management compared to usual care through health centres and haematology units, and for this, we analysed outcomes as a function of case/control status. Specifically, we analysed the time in therapeutic range, percentage of in-range measurements, and clinical complications. We also explored pre- to post-intervention changes in quality of life in the intervention group.

To calculate the percentage of time in days when the INR was in the therapeutic range, we used the Rosendaal test (considering a value <60 % to indicate poor control, based on [26]). Further, we calculated the percentage of INR measurements in range during the 6 months prior to the study and the 6 months of the study, for both intervention and control groups.

The frequency of testing differed between the groups. Patients in the control group continued to be tested at the usual frequency, as scheduled with their usual doctor, while patients in the intervention group (under self-management) were scheduled to self-test every 7 days, in line with the pattern in previous similar studies. The test results of the self-management group were reviewed by a doctor, but no intervention was performed. In the case of controls, their nurse conducted the tests, and their doctor reviewed the results and prescribed their treatment.

We designed a relational database using Microsoft Access 2010 for all the study data. The descriptive and statistical analysis was carried out using R software version 3.1.2 [27].

## Results

**Fig. 1:**
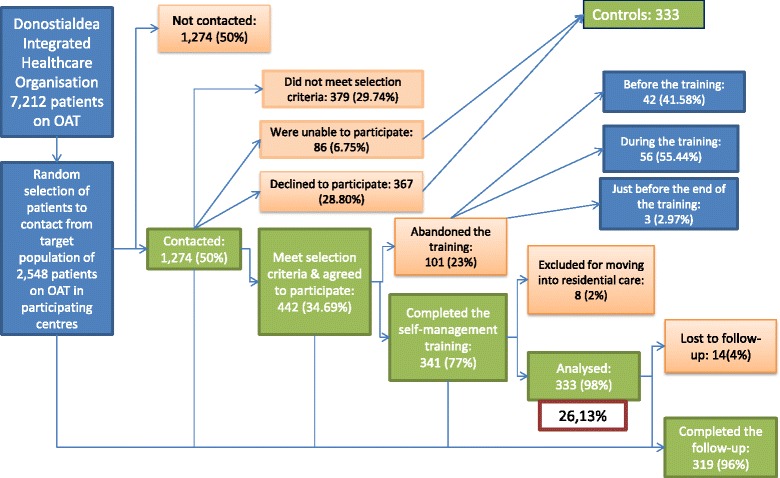
Flow of patients through the study

The study was conducted in the Donostialdea Integrated Healthcare Organisation, which has 21 health centres, with a total of 7,212 patients on OAT (Fig. 1). We selected seven centres with different characteristics in terms of size, and urban or rural settings, among other factors, leaving a target population of 2,548 patients. The number of patients initially selected from each centre was proportional the total eligible population at the centre.

To achieve the desired sample size for the intervention group, we contacted 1,274 patients (50 % of the selected target population), and 442 (35 %) meet selection criteria and agreed to participate in the intervention (Fig. 1). Of this group (*n* = 442) that started the study, 101 patients (22.85 %) did not complete the training process: 42 did not attend the first meeting, while 36 withdrew after the first workshop, 20 after the second workshop and 3 just before the end. Out of the 341 patients who completed the training programme, 8 (2.3 %) were not included in the analysis as they had moved into residential care, although they did not have any problems in terms of participation.

Finally, 333 intervention group patients were included in the analysis (26.13 % of those initially contacted). Out of the 832 individuals that did not take part in the intervention, 367 (29.51 %) declined to participate, 379 (29.74 %) did not met the selection criteria, and 86 (6.75 %) were unable to participate (due to treatment cessation or changes, hospitalisation, pre-surgical admission, moving away from the area or being regularly away, or monitoring in other centres).

There were no significant differences in participation rate by time on OAT, risk factors, the presence of polypharmacy, or whether patients required a caregiver. All 333 patients invited to join the control group agreed.

### Patient characteristics

Among the intervention group participants, 65.5 % were men and the mean age was 73.5 years (Table 2). The recommended INR range was 2.0-3.0 in most cases (79 %), being somewhat higher (2.5-3.5) in the others. The mean reason for OAT was heart arrhythmia due to atrial fibrillation followed by aortic valve prosthesis. Regarding length of treatment, most participants had been on OAT for 4 years or more. Around two-thirds of participants were independent, a third required a caregiver, and 10.5 % required home care. The most common risk factors identified were hypertension, hyperlipaemia and diabetes.

**Table 2:** Summary of patient characteristics at baseline

		Usual monitoring		Self- management		*p*-value
Patients, *n*		333		333		
Sex, *n* (%)	Male	160	(48.0)	218	(65.5)	*p* < 0.0001
	Female	173	(52.0)	115	(34.5)	
Age, mean (SD)	Male	75.7	(9.2)	69.5	(11.9)	*p* < 0.0001
	Female	77.7	(8.5)	73.5	(13.8)	
Reason for OAT, *n* (%)	Heart arrhythmia due to atrial fibrillation	242	(72.7)	221	(66.4)	0.051
	Aortic prosthesis	24	(7.2)	43	(12.9)	
	Deep vein thrombosis	15	(4.5)	23	(6.9)	
	Mitral valve prosthesis	17	(5.1)	17	(5.1)	
	Pulmonary thromboembolism	21	(6.3)	10	(3.0)	
	Cerebrovascular accident	7	(2.1)	5	(1.5)	
	Deficit proteins	0	(0.0)	4	(1.2)	
	Others	3	(0.9)	4	(1.2)	
	Valvular heart disease	3	(0.9)	4	(1.2)	
	Ischemic heart disease	1	(0.3)	2	(0.6)	
INR ranges *n* (%)	1.5–2.5	0	(0.0)	1	(0.3)	0.029
	2.0–3.0	287	(86.2)	264	(79.3)	
	2.5–3.5	46	(13.8)	68	(20.4)	
Length of treatment,	1–3	96	(29.0)	85	(25.7)	0.77
years (%)	4–7	105	(31.7)	106	(32.0)	
	8–10	52	(15.7)	54	(16.3)	
	>10	78	(23.6)	86	(26.0)	
Risk factors,	Arterial hypertension	217	(37.8)	196	(37.9)	0.132
*n* (%)	Diabetes mellitus	85	(14.8)	69	(14.4)	
	Hyperlipaemia	169	(29.5)	180	(34.1)	
	Cancer	50	(9.2)	35	(6.8)	
	Liver disease	6	(1.3)	14	(1.5)	
	Kidney disease	40	(7.4)	32	(5.3)	
Polypharmacy, *n* (%)	1–3 drugs	70	(21.3)	102	(39.5)	*p* < 0.0001
	4–7 drugs	139	(42.6)	156	(37.2)	
	8–12 drugs	108	(30.9)	59	(19.8)	
	13 or more drugs	16	(5.1)	16	(3.5)	
Other,	Independent	226	(67.9)	219	(65.8)	0.621
n (%)	Caregiver	107	(32.1)	114	(34.2)	

Comparing the groups, which were the same size, the controls were somewhat older (mean of 4.2 years) and there was a higher percentage of men (65.5 %) in the intervention group, while the gender distribution was balanced in the control group. Other differences were the distribution of the patients in terms of the number of drugs taken, though all patients in both groups were taking multiple medications, and that more patients under self-management than controls had a slightly higher recommended INR range (2.5–3.5). On the other hand, there were no significant differences between groups in time on OAT, reason for OAT, risk factors, or independence/reliance on a caregiver.

### Intervention group training

Out of the 333 patients included in the self-management intervention, 14 (4.20 %) despite having completed the training successfully did not complete the follow-up for various reasons unrelated to the self-monitoring, such as death, changes in treatment and moving away. Of those who completed the intervention, 16 (4.80 %) found difficulties in learning the knowledge/skills taught in the training programme and required extra support to complete it. Only one person did not achieve the proposed learning objectives.

Overall, nearly all (99.69 %) of the intervention group patients who completed the training and follow-up were considered to be competent in self-management, on the basis that as well as having met the learning objectives of the programme, they met the following criteria: good handling of the coagulometer, lancet, and test strips; good interpretation of results; appropriate decisions on treatment adjustment on more than 95 % of occasions; and correct data entry. Given the negligible proportion of patients not considered to be competent (1 case), we did not carry out a comparative analysis.

### INR control

The mean in-range time in the intervention group (74.4 %) was notably higher than that in controls from other studies (58,6 %) [21]. The mean difference score between the control group and the study measurements was 15,8 % (95 % confidence interval: 5.67, *p* < 0,001).

Over the 6 month study period, the intervention group patients performed 26 INR self-tests and control group patients had 14 tests conducted by clinicians.

Considering the raw data, INR measurements in controls were in range in 63 % of tests, results being 12 % better in the intervention group (75 %; *p* < 0.0001). Further, analysing the individual INR data for each patient, results were similar, 34 % and 24 % of INR measurements being out of range in controls and intervention group patients respectively (*p* < 0.0001).

Analysing the individual percentages of in-range measurements for all patients (Table 3), 58.26 % of patients under usual care and 88.89 % of patients under self-management can be considered to have achieved good INR control (indicated by more than 60 % of measurements lying within range).

**Table 3:** Percentage of INR measurements within the range

% in-range for each patient	Patients under usual monitoring	%	Patients under self-management	%
<50 %	61	18.32	8	2.40
50–55 %	41	12.31	13	3.90
56–60 %	37	11.11	16	4.80
61–65 %	18	5.41	28	8.41
66–70 %	34	10.21	42	12.61
71–75 %	38	11.41	46	13.81
76–80 %	16	4.80	52	15.62
>80 %	88	26.43	128	38.44

### Clinical complications

During the study period, the rate of severe haemorrhage was somewhat higher in the usual care than in the self-management group, and the same was true for acute myocardial infarction, mild haemorrhage, thromboembolism and death; though the differences did not reach significance (Table 4).

**Table 4:** Results of the questionnaires

Item	Baseline	6 months	%	*p*-value
General treatment dissatisfaction	2.27	2.03	−10.57	<0.001
Self-efficacy (in disease management)	4.51	4.93	+9.31	<0.001
Strained social network (psychological stress)	3.17	2.87	−9.46	<0.001
Daily hassles	2.14	1.94	−9.34	0.017
Distress (in social situations)	1.78	1.65	−7.30	0.019

### Quality of life

The quality of life questionnaire administered before and after the intervention was used to explore changes following the initiation of self-management. Pre-intervention results were comparable with those of Blazquez et al. [25]. A total of 415 questionnaires were received from self-management patients, 196 at baseline (58.85 %) and 219 (65.76 %) at the end of the intervention, and the results indicated significant improvements in quality of life.

## Discussion

This study was conceived from a primary care setting to analyse the ability of patients on OAT to self-test and self-manage, as well as assess the clinical impact of this approach, in terms of the control of their treatment and complications.

Almost all patients (99.7 %) who participated in the self-management programme were considered to have acquired the target knowledge/skills. This study demonstrates that patients who attend and complete a self-management programme are successfully able to self-manage, as has been found by other authors, though success rates vary (e.g., Fitzmaurice et al. [10] and De Felipe et al. [28] reporting rates of 57 and 100 % respectively). In our study, 16 patients (4.80 %) found it more difficult than had been expected to achieve the learning objectives, but succeeded with just two additional sessions. Menéndez-Jándula et al. [17] found a considerably higher rate (13 %) of patients requiring extra support. Cayley, WE Jr [18] indicated that between 25 and 90 % of patients may be able to self-test and self-manage, but they did not analyse the underlying reasons; that is, the authors mention some possible barriers but do not assess their relative importance.
  In our study, the main barrier to self-management was related to reluctance of patients to participate, this contributing to reduce the sample analysed to 26.13 % of those contacted. Other researchers have mentioned this issue but did not quantify the extent of the problem [11]. Overall, therefore, nearly three-quarters (73.87 %) of patients wecontacted were not finally included in analysis of the self-management programme, in most cases for personal reasons, reasons given by caregivers or health professionals, or other non-modifiable factors such as hospitalisation, discontinuation of or changes in treatment, moving away or monitoring in other centres. Similar factors have been identified in some other studies [11, 18]. Other authors mention a different range of factors that hinder follow-up of participants, including patient age, medical condition, skills, level of education, willingness, social level, and clinical events, as well as the influence of health professionals [11, 17, 29].
  In our case, the rate of withdrawal during the training period was 23 % of those who initially attended. As reported by other researchers, withdrawal by this type of patients is relatively common, ranging from 17 to 33 % [17].
2. Notably, 96 % of those initially included in the intervention group achieved good self-management during the 6-month study period (in terms of INR measurements, treatment adjustment, and data entry in more than 95 % of the measurements). Only 14 patients (4.20 %) were lost to follow-up, but this was for reasons unrelated to self-management, compared to a figure of 21 % in the study of Menéndez-Jándula et al. [17].
3. Like in previous research [18], in our study, age was not a determinant factor, though self-management patients were a mean of 4 years younger than the controls. Further, in the self-management group, more than half of participants were men (65.5 %), but no differences were observed between groups as a function of aetiology, concurrent illnesses or mental or physical ability [21].
4. Self-management may be considered an acceptable option if we obtain similar results to those obtained with usual care. In fact, comparing data with those monitored through haematology services and health centres, our self-management patients obtained better INR control (in terms of both percentage of in-range measurements compared to controls and time in-range compared to usual values). Over the last 15 years, there have been a growing number of studies involving patients self-managing OAT [4, 5, 7, 10, 11, 15–18, 30], several authors reporting similar results to ours [4, 8, 10, 31].
5. Moreover, we found lower rates of clinical complications (serious or mild), deaths, and admissions and a better clinical course in patients under self-management, confirming observations of Heneghan et al. [29] and Bloomfield et al. [32]. Analysing the types of complications, we observed that many of them occurred concurrent with other health problems (rectorrhagia with intestinal lesions and haemorrhoids, haematuria with prostate, kidney or bladder cancer, metrorrhagia with cervical or womb cancer), and we propose that these should be the focus of separate research. On the other hand, some other types of bleeding such as epistaxis, gum bleeding, and microhaematuria may be more closely related to OAT, since they did not occur together with other aetiologies. We have not, however, quantified these data.
6. In our study, self-management patients reported high levels of personal satisfaction, as reflected in the quality of life results (Table 5), confirming the observations of other authors [11, 17, 29, 33].

**Table 5:** Percentage of time in therapeutic INR range in patients under self-management

Percentage of time in-range for each patient	Patients under self-management	%
<50 %	31	9.31
50–55 %	26	7.81
56–60 %	36	10.81
61–65 %	30	9.01
66–70 %	45	13.51
71–75 %	39	11.71
76–80 %	41	12.31
>80 %	85	25.53

There is growing evidence that self-management is not just another option, rather it is a system that actually improves outcomes in patients, in terms of INR control, clinical complications, and quality of life [11, 17, 29]. We have not conducted cost analysis, but plan to do so in the near future.
7. Remote monitoring of patients was key to the study, given that self-management carries a certain level of risk for patients but with telemonitoring this risk could be managed. Notably, telemonitoring was only used for checking progress and was not interventionist in nature.

Patients were asked to use a website to submit data about their measurements, the decisions they took depending on their results, and any clinical events. A total of 93 % managed to use the website (by themselves, or with help, from relatives, friends, caregivers, or a community centre, among other strategies), the remaining patients reporting their data by phone.

Researchers reviewed the data submitted by patients every day and noted the actions taken following each test, to assess the quality of self-management and detect any possible erroneous actions that could pose a risk to the patient’s health. Patients were contacted to resolve concerns in relation to 2 % of measurements, but in no cases was it considered necessary to intervene or modify the actions taken by patients.

The 24-h telephone helpline was maintained throughout the follow-up period, to resolve clinical or technical concerns. It was used a mean of 0.6 times per patient during the 6 month period. Of the total of 76 calls received, 32 (42 %) were related to administrative issues and 25 (32.9 %) to technical problems, while 19 concerned self-management itself, all of these calls occurring in the first month of the study.

These channels of communication with patients during the study period were found to be useful, the web data making it possible to immediately assess patients’ actions to avoid serious risks, while clinical and technical problems were resolved over the telephone, and though there were relatively few such problems, the helpline strengthened patients’ confidence. These resources have been important to safeguard patient safety while avoiding regular contact between researchers and patients that would otherwise have been necessary.

We have found no other studies that have employed such an approach. Telemonitoring has tended to be used as a tool for doctor-patient communication but not for self-managed patients. We believe that these two channels of communication combined make a great tool, both highly practical and feasible, and that their use could be widened to monitoring of patients with other chronic illnesses.

There was also good participation from health professionals, doctors and nurses at the health centres, despite the fact that at the outset we thought this might have been a weak point of the study. Their support was very valuable. We consider that the information they were given about the study, their supervision, the initial clinical session and open channels of communication were essential for the positive attitudes shown. A possible future line of research would be to explore strategies for changing attitudes of patients, caregivers and health professionals [19], in order to assess the possibility of increasing the number of patients under this type of care. Further, there is a need for more research to establish the relationship between OAT and various types of complications, analysing their causes.

Strengths of the study

This was a randomised multicentre study conducted in primary care with no limits on age, medical condition, mental or physical status, drawing patients from small, medium and large health centres in rural and urban settings, with no conditioning economic factors and with support from the Carlos III Health Institute, Osakidetza, and Roche.

Weaknesses

Patients were recruited progressively and hence the training was not carried out at the same time for all participants and the data for controls was collected over a different time period. Further, given the design of the sample, the intervention group only contained individuals who wanted to participate.

## Conclusions

Patients who are fit and willing and/or who have a suitable and willing caregiver are able to self-manage OAT, regardless of age, reason for the OAT, and the presence of multimorbidity, polypharmacy or other risk factors. Our results underline the potential of self-management across the full spectrum of patients, those under this type of management not only matching but improving on the level of INR control achieved under usual care.

The results obtained in this study demonstrate the ability of motivated patients to self-manage, levels of control being not only as good as but even better than those receiving usual care. Self-management is also associated with an improvement in patient quality of life. The fact that self-management improves control and other outcomes in patients on OAT should strengthen our interest in the use of this approach in other chronic illnesses. A possible future avenue of research would be to explore ways of changing attitudes in patients, caregivers and clinicians [18] seeking to increase the proportion of patients who engage in self-management. Further research is also required into the economic and organisational impact of self-management on health services.

## Abbreviations

INR: International normalized ratio; OAT: Oral anticoagulation therapy.

## Figures and Tables

**Fig. 2:**
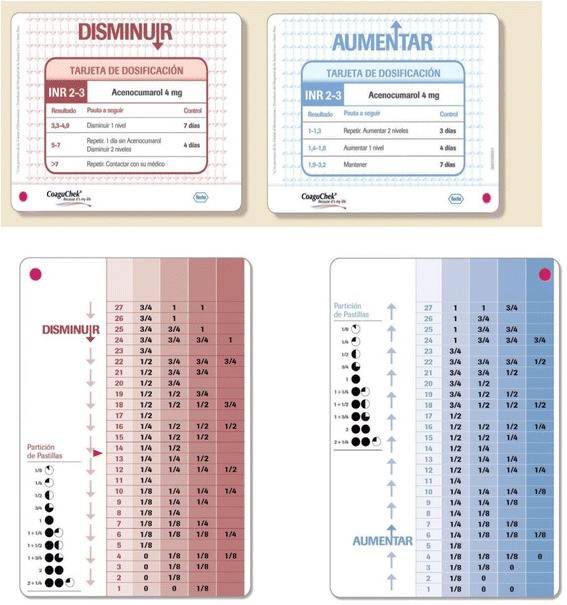
Tables used by AC OAT patients to adjust the dose depending on the results of the INR

## Appendix I

### Training

*First workshop*: In a session lasting 1 h, groups of four people were provided with information and training related to the characteristics of their condition and treatment. At the end of the session, every patient was requested to sign an informed consent form. Those who agreed continued in the study, while those who declined were replaced by other patients.

*Second workshop*: Held the following day and also lasting 1 h, this session focused on the use of the coagulometer (device for determining INR) and use the tables for self-adjusting their treatment. They were also taught how to enter their data into a form on a website, allowing healthcare professionals leading the study to monitor their progress. They were requested to complete a questionnaire on the quality of life of patients on OAT.

In this workshop, patients practiced what they would have to do later by themselves:

handling of the coagulometer, completion of INR measurements, simulation of dose adjustment, and data entry on the website: https://autocontrol.taonet.es/tao.

Patients, with a username and password, were able to access their data and enter data on the results and observations every time they measured their INR. The principal investigator of the project accessed the data entered by all the patients every day, to check on the progress of the self-management of each patient, assessing both the INR data and the clinical notes of patients, aiming to avoid serious errors in the process.

*Third workshop*: This was held 1 week after the first workshop. Patients carried out the test in their own homes and then attended the workshop. Patients’ learning was checked and the content of previous sessions was reviewed. Any remaining doubts were addressed.

If patients were considered to have met the learning objectives (successfully using the coagulometer, obtaining a drop of blood, applying the tables, and communicating the results), they were provided with all the materials necessary for performing self-testing over the 6-month study period and were given an appointment for 3 weeks later. If not, they were given extra sessions, after which those considered ready for self-management were provided with the aforementioned materials and given an follow-up appointment at 3 weeks. If we considered that patients had not learned enough, even with extra support, agreement was reached that they would not participate in the study.

*Fourth workshop*: At 1 month after the first workshop, we checked what patients had done during the month of self-management, and INR measurements taken using the coagulometer data were downloaded. We addressed any remaining problems or queries and finally certified that they had completed the training satisfactorily.

*Fifth workshop*: After 6 months, patients were contacted by phone to arrange a final session. We collected the data from the coagulometer and transferred them to the computer software, cross-checking with the data submitted by patients themselves.

Lastly, patients were requested to complete the questionnaire on the quality of life of patients on OAT for a second time. We thanked patients for their participation, giving them a diploma and declaring that the study had been completed.
